# Estrogenic botanical supplements, health-related quality of life, fatigue, and hormone-related symptoms in breast cancer survivors: a HEAL study report

**DOI:** 10.1186/1472-6882-11-109

**Published:** 2011-11-08

**Authors:** Huiyan Ma, Jane Sullivan-Halley, Ashley W Smith, Marian L Neuhouser, Catherine M Alfano, Kathleen Meeske, Stephanie M George, Anne McTiernan, Roberta McKean-Cowdin, Kathy B Baumgartner, Rachel Ballard-Barbash, Leslie Bernstein

**Affiliations:** 1 Division of Cancer Etiology, Department of Population Sciences, Beckman Research Institute, City of Hope, Duarte, CA 91010, USA; 2 Division of Cancer Control and Population Sciences, National Cancer Institute, Bethesda, MD 20892, USA; 3 Fred Hutchinson Cancer Research Center, Seattle, WA 98109, USA; 4 Childrens Center for Cancer and Blood Diseases, Children's Hospital Los Angeles, Los Angeles, CA 90027, USA; 5 Division of Cancer Epidemiology and Genetics, National Cancer Institute, Bethesda, MD 20892, USA; 6 Department of Preventive Medicine, Keck School of Medicine, University of Southern California, Los Angeles, CA 90089, USA; 7 Epidemiology and Clinical Investigation Sciences, School of Public Health & Information Sciences, University of Louisville, Louisville, KY 40202, USA

## Abstract

**Background:**

It remains unclear whether estrogenic botanical supplement (EBS) use influences breast cancer survivors' health-related outcomes.

**Methods:**

We examined the associations of EBS use with health-related quality of life (HRQOL), with fatigue, and with 15 hormone-related symptoms such as hot flashes and night sweats among 767 breast cancer survivors participating in the Health, Eating, Activity, and Lifestyle (HEAL) Study. HRQOL was measured by the Medical Outcomes Study short form-36 physical and mental component scale summary score. Fatigue was measured by the Revised-Piper Fatigue Scale score.

**Results:**

Neither overall EBS use nor the number of EBS types used was associated with HRQOL, fatigue, or hormone-related symptoms. However, comparisons of those using each specific type of EBS with non-EBS users revealed the following associations. Soy supplements users were more likely to have a better physical health summary score (odds ratio [OR] = 1.66, 95% confidence interval [CI] = 1.02-2.70). Flaxseed oil users were more likely to have a better mental health summary score (OR = 1.76, 95% CI = 1.05-2.94). Ginseng users were more likely to report severe fatigue and several hormone-related symptoms (all ORs ≥ 1.7 and all 95% CIs exclude 1). Red clover users were less likely to report weight gain, night sweats, and difficulty concentrating (all OR approximately 0.4 and all 95% CIs exclude 1). Alfalfa users were less likely to experience sleep interruption (OR = 0.28, 95% CI = 0.12-0.68). Dehydroepiandrosterone users were less likely to have hot flashes (OR = 0.33, 95% CI = 0.14-0.82).

**Conclusions:**

Our findings indicate that several specific types of EBS might have important influences on a woman's various aspects of quality of life, but further verification is necessary.

## Background

Breast cancer survivors frequently use complementary/alternative medicines (CAM) such as estrogenic botanical supplements (EBS) in hopes of improving their health-related quality of life (HRQOL), boosting their sense of well-being, and alleviating the side-effects of conventional therapies [1,2]. However, no published data exist regarding the associations of EBS use with HRQOL, fatigue, or symptoms often characterized by a deficit of estrogen.

Botanical supplements are plant parts such as bark, leaves, stems, roots, flowers, fruits, seeds and berries or their extracts that are sold as pills, capsules or extracts [3]. EBS refer specifically to botanical supplements with phytoestrogenic components that may have weak estrogenic properties, directly alter estrogen levels, or function directly on receptors in different organs as either pro- or anti-estrogens [4]. EBS effects may differ depending upon concentration or the different components within different plant source [5].

High estrogen levels are well-documented risk factors for breast cancer [6-8] and anti-estrogenic therapy is a mainstay of adjuvant treatment for breast cancer [9]. Studies of the EBS impact on endogenous estrogen levels are mixed, showing increased [10], reduced [4], or no association with circulating levels of estrogen [11,12]. We previously showed that, among postmenopausal breast cancer survivors participating in the Health, Eating, Activity, and Lifestyle (HEAL) Study, EBS users had lower estrone, estradiol and free estradiol levels than non-EBS users [13].

Previous epidemiologic studies among breast cancer survivors have examined the associations of CAM or botanical supplements with HRQOL and hormone-related symptoms showing that use was associated with poorer mental health function [14-17], poorer physical health function [17,18], and hormone-related symptoms listed by the National Surgical Adjuvant Breast and Bowel Project Breast Cancer Prevention Trial [14,19]. However, none of these studies specifically focused on EBS use.

Here, we examine a new hypothesis to determine whether EBS use (overall, by number of EBS types, or by specific type used) is associated with HRQOL, fatigue, or 15 hormone-related symptoms among breast cancer survivors who had survived an average of 30 months after their first primary *in situ *or invasive breast cancer diagnosis.

## Methods

### Study population

Women participating in the HEAL Study, a multicenter, multiethnic, prospective study of 1, 183 women diagnosed with first primary *in situ *or invasive breast cancer between 1994 and 1999, provided the data for this analysis [20,21]. Breast cancer survivors were recruited within 12 months (mean = 6.1 months) following their breast cancer diagnosis through the Surveillance Epidemiology and End Results (SEER) registries in New Mexico (n = 615), Western Washington (n = 202), and Los Angeles County (n = 366).

Of 1, 183 women who completed the baseline survey, 944 (80%) participated in a second assessment approximately 30 months (mean = 30.4 months) after diagnosis. Of the non-participants, 44 were deceased, 104 refused to participate, 55 could not be located, 17 could not be contacted, and 19 were too ill.

Of the original 1, 183 women, 858 (73%) completed a third assessment approximately 40 months (mean = 40.6 months) after diagnosis. Of the non-participants, 75 were deceased, 140 refused to participate, 49 could not be located, 50 could not be contacted, and 11 were too ill.

Among 829 women who completed all three surveys, we excluded 41 women who had subsequent recurrences or new primaries before their 30-month assessment, because these subsequent events and corresponding treatments might influence both EBS use and multiple health-related outcomes of interest. This yielded a preliminary analytic sample of 788 women.

All participants provided informed consent before each survey. The Institutional Review Boards at participating centers approved study protocols, in accordance with assurances filed with and approved by the United States Department of Health and Human Services.

### Data collection

Diagnosis date and stage of breast cancer were based on SEER data. Treatment data (surgery, radiation therapy, and chemotherapy) were abstracted from medical records or, when unavailable, from SEER data.

Baseline in-person interviews in New Mexico and Los Angeles and self-administered questionnaires in Washington provided data on education, race/ethnicity, birth date, and height (measured in clinics in Washington and New Mexico and self-reported in Los Angeles).

Participants were asked about EBS use in the 30-month assessment: ''Since your cancer diagnosis have you taken any herbal or alternative remedies?'' Participants who answered yes were given a list of 34 commonly used botanical or herbal supplements and asked to indicate which, if any, of these supplements they used. An ''Other'' category was used to collect supplements that were not on the list; 94 distinct supplements were recorded via this open-ended question.

We reviewed all botanical-type supplements for evidence of estrogenicity using the Physician's Desk Reference for Herbal Medicines (PDR-H) [22]. For some supplements, evidence of estrogenicity was not clearly defined; however, we considered a supplement as estrogenic if at least one study (in vitro, animal or human) was cited that showed estrogenic properties. For supplements not found in the PDR-H, we consulted Herb-Drug Interactions in Oncology (HDIO) [23] or the Natural Medicines Comprehensive Database (NMCD) [24]. A majority of supplements (90/128, 70%) were identified in the PDR-H, six supplements (5%) in HDIO and the remaining 32 supplements (25%) in the NMCD. Of the 128 botanical/herbal supplements used by HEAL participants, 19 had estrogenic properties based on our definition, which included soy supplements, ginseng, flaxseed oil, black cohosh [Cimicifuga racemosa], yam, dong quai, red clover, licorice, alfalfa, cat's claw, dehydroepiandrosterone (DHEA), astragalus, boron, burdock root, fo ti tieng, nettles, saw palmetto, turmeric, and a combination supplement (containing soy, black cohosh, licorice, and dong quai).

Information was collected during the 30-month survey on menopausal status, hormone therapy (HT) use, physical activity, tamoxifen use, and weight. Menopausal status was determined by age, menstrual status in the past year, HT use, number of ovaries and history of hysterectomy using an algorithm that assigned women into premenopausal, postmenopausal or unclassifiable menopausal status. Physical activity was measured using a version of the Modifiable Activity Questionnaire [25] adapted for this study; the type, duration, and frequency of 20 activities (e.g., walking, jogging, aerobics, tennis) during the past year were assessed. The MET intensity of each activity was classified as light, moderate, or vigorous based on its rating in the Compendium of Physical Activities [26]. MET hours per week of sports activity and recreational physical activity were combined into a total score and categorized (0, 0.1-8.9, ≥ 9 MET hours/week) based on prior analyses in this cohort [27]. Weight was measured in clinics in Western Washington and New Mexico and during interviews in Los Angeles. Body mass index (kg/m^2^) was based on weight measured at the 30-month survey and height collected at the baseline survey.

In the 30-month survey, we inquired whether participants had been diagnosed by a physician with any of 18 chronic medical conditions (e.g., angina, arthritis, osteoporosis, chronic lung disease, diabetes, other cancers) and, if yes, whether that condition limited current activities of daily living. Medical comorbidity was calculated as the number of conditions reported as limiting current activities of daily living. We measured diet using a 122-item self-administered food-frequency questionnaire (FFQ) developed and validated for the Women's Health Initiative [28]. Isoflavones (mg/day) from soy-containing foods on the FFQ were estimated as the sum of dietary genestein and dietary daidzein.

We assessed participants' HRQOL using the Medical Outcomes Study short form-36 (SF-36) questionnaire during the 40-month survey [29]. This tool contains 36 items and provides a physical component summary (PCS) scale and a mental component summary (MCS) scale. The two SF-36 summary scales are both valid and reliable [29-31]. These scales were scored in reference to a normal population (the 1998 general US population, standard form) with a transformed mean of 50, and a standard deviation of 10 [32]. Higher scores on each scale represent better QOL; summary scale scores above 50 indicate that QOL is above average.

We also used the Revised-Piper Fatigue Scale to assess fatigue at the 40-month assessment [33]. This scale contains 22 items, measures four dimensions of subjective fatigue (behavioral, sensory, cognitive/mood and affective), and provides an overall total fatigue score, with higher scores indicating a greater degree of fatigue. We used an adapted version of the Revised-Piper Fatigue Scale score [34] that asks survivors to rate their fatigue over the past month rather than the past week to minimize the effect of acute situational events and to enhance our assessment of the survivor's general state of fatigue. The Revised-Piper Fatigue Scale score has demonstrated acceptable internal consistency, content validity and concurrent criterion validity with adult cancer survivors [35-37].

We used a modified 15-symptom version of the National Surgical Adjuvant Breast and Bowel Project Breast Cancer Prevention Trial checklist [19,38] to collect information on hormone-related symptoms at the 40-month survey asking women to indicate how much they were bothered by any of the problems during the past year only. The symptoms surveyed were hot flashes, difficulty with bladder control when laughing or crying, difficulty with bladder control at other times such as when coughing or sneezing, vaginal discharge, genital itching/irritation, pain with intercourse, breast sensitivity/tenderness, weight gain, unhappy with bodily appearance, forgetfulness, tendency to take naps/stay in bed, night sweats, difficulty concentrating, easily distracted, interrupted sleep, irritability and mood swings. Response options for the amount bothered by the symptoms were: "Not at all", "Slightly", "Moderately", "Quite a bit", "Extremely". Women who responded "Not at all" for a given symptom were considered not to have experienced that symptom.

### Statistical analysis

We compared EBS users with non-users using Pearson χ^2 ^tests to evaluate differences in the frequency distributions of categorical variables and *t *tests to evaluate differences in means of continuous variables.

We treated the PCS scores, MCS scores, and total fatigue scores as continuous variables and also dichotomized the scores (low, high). Both PCS and MCS scores were dichotomized at the standardized mean of 50 (< 50, ≥ 50) [32], which has been used previously [39,40]. The cut point for total fatigue scores was based on prior work in this cohort (< 4, ≥ 4) [41].

Multivariable linear regression models were fit to examine whether overall EBS use (no, yes), number of EBS types (none, 1 type, ≥ 2 types), or specific type of EBS (soy supplements, ginseng, flaxseed oil, black cohosh, yam, dong quai, red clover, licorice, alfalfa, cat's claw, DHEA, or other EBS) was associated with continuous values of the PCS scores, MCS scores, and total fatigue scores. We fit multivariable unconditional logistic regression models using dichotomous outcome measures to determine whether EBS use was associated with high PCS (≥ 50), MCS (≥ 50), or total fatigue scores (≥ 4). Odds ratios (ORs) and 95% confidence intervals (CIs) for EBS use were estimated from these models. We also fit multivariable unconditional logistic regression models, to examine whether EBS use was associated with each hormone-related symptom (yes vs. no).

All multivariable regression models adjusted for variables that were statistically significantly different when comparing EBS users to non-users in Table 1: education, age at diagnosis, medicial comorbidity, MET hours per week of sports activity and recreational physical activity from 30-month interview, a combined variable for menopause and HT use, and isoflavones from soy-containing foods. In the analyses of individual types of EBS, we adjusted for all other types of EBS used.

**Table 1:** Study population characteristics by estrogenic botanical supplement (EBS) use

	No. non-EBS users (%)	No. EBS users (%)	*P *value^a^
	N = 464	N = 303	
Study site			*0.12*
Western Washington	90 (19.4)	70 (23.1)	
New Mexico	259 (55.8)	146 (48.2)	
Los Angeles County	115 (24.8)	87 (28.7)	
Race			*0.63*
Non-Hispanic white	281 (60.6)	173 (57.1)	
African-American	115 (24.8)	88 (29.0)	
Hispanic	53 (11.4)	33 (10.9)	
Others	15 (3.2)	9 (3.0)	
Education			*< 0.0001*
≤ High school	143 (30.8)	51 (16.8)	
Technical school or some college	154 (33.2)	126 (41.6)	
College graduate	167 (36.0)	126 (41.6)	
Mean age at diagnosis (SD^b^), years	56.0 (10.6)	52.5 (8.7)	*< 0.0001*^c^
Stage at diagnosis			*0.41*
In situ	110 (23.7)	60 (19.8)	
Localized	257 (55.4)	173 (57.1)	
Regional	97 (20.9)	70 (23.1)	
Breast cancer treatment			*0.43*
No radiation and no chemotherapy	155 (33.4)	86 (28.4)	
Radiation only	171 (36.9)	116 (38.3)	
Chemotherapy only	45 (9.7)	29 (9.6)	
Radiation and chemotherapy	93 (20.0)	72 (23.8)	
Tamoxifen use			*0.24*
No	248 (53.5)	175 (57.8)	
Yes	216 (46.6)	128 (42.2)	
Medical comorbidity			*0.007*
None	332 (71.6)	243 (80.2)	
1 or more condition(s)	132 (28.5)	60 (19.8)	
Activity level within the past year of 30-month interview from sports/recreation (MET hours/week)			*< 0.0001*
0	93 (20.0)	33 (10.9)	
0.1-8.9	201 (43.3)	115 (38.0)	
≥ 9	170 (36.6)	155 (51.2)	
Menopausal status at 30-month interview			*0.01*
Premenopausal	74 (16.0)	68 (22.4)	
Postmenopausal			
Never HT^d ^after diagnosis	193 (41.6)	135 (44.6)	
Ever HT^d ^after diagnosis	172 (37.1)	80 (26.4)	
Unknown	25 (5.4)	20 (6.6)	
Body mass index at 30-month interview (kg/m^2^)			*0.13*
< 25	180 (38.8)	128 (42.2)	
25-29	133 (28.7)	97 (32.0)	
≥ 30	151 (32.5)	78 (25.7)	
Mean isoflavones from soy-containing foods (SD^b^), mg/day	1.9 (9.4)	4.0 (9.9)	*0.002*^c^

^a^*P*-value ascertained from Pearson χ^2 ^test, except where otherwise noted. ^b^SD, standard deviation. ^c^*P*-value from t test. ^d^HT, hormone therapy.

To use a constant sample size, we excluded 21 women who were missing information on education (n = 1), medical comorbidity (n = 1), MET hours per week of sports activity and recreational physical activity from 30-month interview (n = 3), isoflavones from soy-containing foods (n = 12), HRQOL scores (n = 1), or fatigue scores (n = 3). The 21 women did not differ from the remaining 767 women on age at diagnosis, breast cancer stage, or treatment for breast cancer.

In reporting results from regression analyses, we considered a two-sided *P *value ≤ 0.05 as statistically significant. We did not adjust *P *values for multiple comparisons as these analyses were considered as exploratory [42]. All analyses were performed using the SAS statistical package (Version 9.2, SAS Institute, Cary, NC).

## Results

### Characteristics of EBS users and non-users

EBS was used by 39.5% of women after their breast cancer diagnoses, including 18.4% who used only one type and 21.1% who used two or more EBS types. Soy supplements (16.6%), ginseng (13.4%), and flaxseed oil (13.0%) were the most commonly used EBS types.

EBS users were more educated (*P*_χ_^2 ^< 0.0001), younger at diagnosis (*P*_t-test _< 0.0001), more physically active (*P*_χ_^2 ^< 0.0001), less likely to have medical comorbidity (*P*_χ_^2 ^= 0.007), more likely to be premenopausal (*P*_χ_^2 ^= 0.01), and more likely to consume isoflavones from soy-containing foods (*P*_t-test _= 0.002) than non-EBS users (Table 1).

### EBS use and HRQOL

Neither overall EBS use nor number of EBS types used was associated with continuous or dichotomous HRQOL scores (Table 2). However, a statistically non-significant positive association was observed between soy supplement use and continuous PCS scores (*P *= 0.08). Soy supplement users had 66% greater odds (OR = 1.66, 95% CI = 1.02-2.70) of a high (≥ 50) PCS score; but no association was observed with MCS score. Ginseng use was negatively associated with the continuous PCS score (*P *= 0.008); ginseng users has 32% decreased odds of a high (≥ 50) PCS score (OR = 0.68, 95% CI = 0.40-1.15); no association was observed with MCS score. The use of flaxseed oil was not associated with continuous or dichotomous PCS score. However, the use of flaxseed oil was statistically non-significantly positively associated with the continuous MCS score (*P *= 0.06). Flaxseed oil users had 76% greater odds (OR = 1.76, 95% CI = 1.05-2.94) of a high (≥ 50) MCS score than non-EBS users.

**Table 2:** The association between estrogenic botanical supplement (EBS) use and health-related quality of life (HRQOL) score

	Association with continuous HRQOL score			Association with better HRQOL (≥ 50)		
	
	No.	Adjusted regression coefficient (standard error)	*P *value	No. with low score < 50	No. with high score ≥ 50	Adjusted OR (95% CI)
Association with physical component summary (PCS)						
**Ever used EBS after diagnosis^a^**						
No	464			228	236	1.00
Yes	303	-1.27 (0.67)	*0.06*	142	161	0.84 (0.60-1.18)
						
**By number of EBS used^a^**						
1	141	-1.56 (0.86)	*0.07*	66	75	0.83 (0.54-1.28)
≥ 2	162	-0.99 (0.84)	*0.24*	76	86	0.85 (0.56-1.28)
						
**By type of EBS used^a, b^**						
Soy supplements	127	1.63 (0.93)	*0.08*	52	75	1.66 (1.02-2.70)
Ginseng	103	-2.77 (1.04)	*0.008*	55	48	0.68 (0.40-1.15)
Flaxseed oil	100	0.41 (1.07)	*0.70*	41	59	1.20 (0.68-2.09)
Black cohosh	68	-0.70 (1.31)	*0.59*	35	33	0.66 (0.35-1.27)
Yam	47	1.13 (1.51)	*0.45*	19	28	1.52 (0.70-3.27)
Dong quai	39	-1.17 (1.65)	*0.48*	21	18	0.70 (0.31-1.61)
Red clover	38	-1.40 (1.70)	*0.41*	22	16	0.61 (0.26-1.41)
Licorice	37	1.37 (1.62)	*0.40*	17	20	1.50 (0.64-3.47)
Alfalfa	31	-0.86 (1.76)	*0.62*	19	12	0.65 (0.26-1.60)
Cat's claw	24	-2.38 (2.00)	*0.23*	13	11	0.80 (0.28-2.28)
DHEA	24	-1.65 (1.85)	*0.37*	16	8	0.40 (0.15-1.07)
Other EBS	34	1.26 (1.67)	*0.45*	16	18	1.17 (0.50-2.76)
						
Association with mental component summary (MCS)						
**Ever used EBS after diagnosis^a^**						
No	464			200	264	1.00
Yes	303	0.57 (0.80)	*0.48*	122	181	1.16 (0.85-1.57)
						
**By number of EBS used^a^**						
1	141	0.80 (1.02)	*0.43*	58	83	1.09 (0.74-1.61)
≥ 2	162	0.35 (1.00)	*0.73*	64	98	1.23 (0.84-1.79)
						
**By type of EBS used^a, b^**						
Soy supplements	127	1.06 (1.11)	*0.34*	44	83	1.42 (0.92-2.20)
Ginseng	103	-0.80 (1.24)	*0.52*	44	59	0.88 (0.55-1.42)
Flaxseed oil	100	2.39 (1.28)	*0.06*	33	67	1.76 (1.05-2.94)
Black cohosh	68	-0.50 (1.57)	*0.75*	28	40	0.94 (0.52-1.73)
Yam	47	-0.36 (1.81)	*0.84*	18	29	1.14 (0.56-2.31)
Dong quai	39	1.28 (1.97)	*0.52*	16	23	0.98 (0.46-2.11)
Red clover	38	0.75 (2.04)	*0.71*	14	24	1.15 (0.51-2.57)
Licorice	37	-1.09 (1.95)	*0.57*	16	21	0.79 (0.37-1.67)
Alfalfa	31	-0.11 (2.10)	*0.96*	13	18	0.89 (0.40-2.00)
Cat's claw	24	-3.07 (2.40)	*0.20*	12	12	0.52 (0.21-1.30)
DHEA	24	1.49 (2.22)	*0.50*	10	14	0.93 (0.40-2.21)
Other EBS	34	0.19 (2.00)	*0.93*	12	22	1.25 (0.57-2.75)

**^a^**Adjusted for education, age at diagnosis, medical comorbidity, activity level within the past year of 30-month interview, a combined variable for menopause and hormone therapy, isoflavones from soy-containing foods. **^b^**Additionally, all types mutually adjusted.

### EBS use and total fatigue score

Neither overall EBS use nor number of EBS types used was associated with fatigue in the linear or logistic regression analyses (Table 3). Ginseng use was associated with greater fatigue measured continuously (*P *= 0.002) as well as in the dichotomous form (OR = 1.70, 95% CI = 1.04-2.76).

**Table 3:** The association between estrogenic botanical supplement (EBS) use and total fatigue score

	Association with continuous total fatigue score			Association with severe fatigue (≥ 4)		
	
	**No**.	Adjusted regression coefficient (standard error)	*P *value	No. with PFS < 4	No. with PFS ≥ 4	Adjusted OR (95% CI)
**Ever used EBS after diagnosis^a^**						
No	464			280	184	1.00
Yes	303	0.13 (0.17)	*0.44*	179	124	1.06 (0.78-1.45)
						
**By number of EBS used^a^**						
1	141	0.11 (0.21)	*0.59*	86	55	0.98 (0.66-1.46)
≥ 2	162	0.14 (0.21)	*0.50*	93	69	1.14 (0.78-1.69)
						
**By type of EBS used^a, b^**						
Soy supplements	127	0.07 (0.23)	*0.75*	75	52	1.16 (0.74-1.80)
Ginseng	103	0.81 (0.26)	*0.002*	54	49	1.70 (1.04-2.76)
Flaxseed oil	100	-0.22 (0.26)	*0.40*	66	34	0.66 (0.39-1.12)
Black cohosh	68	-0.17 (0.32)	*0.60*	43	25	0.78 (0.41-1.46)
Yam	47	-0.003 (0.37)	*0.99*	30	17	0.94 (0.45-1.96)
Dong quai	39	-0.25 (0.40)	*0.53*	24	15	0.83 (0.38-1.81)
Red clover	38	-0.60 (0.42)	*0.15*	26	12	0.52 (0.22-1.21)
Licorice	37	0.07 (0.40)	*0.85*	21	16	1.41 (0.65-3.06)
Alfalfa	31	-0.39 (0.43)	*0.36*	21	10	0.72 (0.30-1.71)
Cat's claw	24	0.79 (0.49)	*0.11*	12	12	2.03 (0.76-5.42)
DHEA	24	0.27 (0.45)	*0.55*	11	13	1.96 (0.82-4.70)
Other EBS	34	-0.57 (0.41)	*0.16*	22	12	0.79 (0.35-1.77)

**^a^**Adjusted for education, age at diagnosis, medical comorbidity, activity level within the past year of 30-month interview, a combined variable for menopause and hormone therapy, isoflavones from soy-containing foods. **^b^**Additionally, all types mutually adjusted.

### EBS use and hormone-related symptoms

Although we examined the potential associations for all the 15 symptoms with overall EBS use, number of EBS types, and each specific EBS, we observed no associations with overall EBS use, number of EBS types, or the majority of specific EBS. We limited presentation of results to four specific EBS types (ginseng, red clover, alfalfa, and DHEA) and the 10 symptoms where we observed at least one statistically significant association (Table 4). Ginseng use was positively associated with vaginal discharge, unhappiness with bodily appearance, forgetfulness, tendency to take naps or stay in bed, and irritability and mood swings (all ORs > 1.8 and all 95% CIs exclude 1). Red clover users were less likely to report weight gain, night sweats, and difficulty concentrating (all OR approximately 0.4 and all 95% CIs exclude 1). Alfalfa users were less likely than non-supplement users to have interrupted sleep (OR = 0.28, 95% CI = 0.12-0.68). DHEA users were less likely than non-supplement users to have hot flashes (OR = 0.33, 95% CI = 0.14-0.82).

**Table 4:** Adjusted^a ^OR (95% CI) for the association between estrogenic botanical supplement use and hormone-related symptoms during last years of 30-month survey

	Ginseng			Red Clover			Alfalfa			DHEA		
	**No**	**Yes**	**Adjusted^a ^OR (95% CI)**	**No**	**Yes**	**Adjusted^a ^OR (95% CI)**	**No**	**Yes**	**Adjusted^a ^OR (95% CI)**	**No**	**Yes**	**Adjusted^a ^OR (95% CI)**

Hot flashes												
No	179	19	1.00	191	7	1.00	187	11	1.00	187	11	1.00
Yes	485	84	1.41 (0.76-2.63)	538	31	0.99 (0.34-2.85)	549	20	0.43 (0.17-1.08)	556	13	0.33 (0.14-0.82)
Vaginal discharge												
No	427	53	1.00	457	23	1.00	460	20	1.00	466	14	1.00
Yes	237	50	1.81 (1.12-2.93)	272	15	0.92 (0.42-2.05)	276	11	0.94 (0.41-2.20)	277	10	1.15 (0.48-2.73)
Weight gain												
No	234	37	1.00	253	18	1.00	257	14	1.00	262	9	1.00
Yes	430	66	0.94 (0.57-1.55)	476	20	0.41 (0.19-0.91)	479	17	0.76 (0.33-1.76)	481	15	0.88 (0.36-2.11)
Unhappy with bodily appearance												
No	221	17	1.00	225	13	1.00	226	12	1.00	230	8	1.00
Yes	443	86	2.58 (1.38-4.81)	504	25	0.49 (0.21-1.17)	510	19	0.67 (0.28-1.60)	513	16	0.76 (0.30-1.93)
Forgetfulness												
No	147	13	1.00	153	7	1.00	154	6	1.00	156	4	1.00
Yes	517	90	2.19 (1.11-4.33)	576	31	1.09 (0.40-2.98)	582	25	1.09 (0.39-3.02)	587	20	1.38 (0.43-4.42)
Tendency to take naps, stay in bed												
No	326	39	1.00	346	19	1.00	352	13	1.00	357	8	1.00
Yes	338	64	2.24 (1.35-3.72)	383	19	1.20 (0.54-2.70)	384	18	1.37 (0.59-3.18)	386	16	1.96 (0.79-4.87)
Night sweats												
No	272	31	1.00	285	18	1.00	288	15	1.00	293	10	1.00
Yes	392	72	1.42 (0.84-2.39)	444	20	0.40 (0.18-0.90)	448	16	0.66 (0.29-1.53)	450	14	0.82 (0.34-1.97)
Difficulty concentrating												
No	268	34	1.00	282	20	1.00	292	10	1.00	293	9	1.00
Yes	396	69	1.51 (0.91-2.50)	447	18	0.41 (0.18-0.90)	444	21	1.84 (0.77-4.41)	450	15	1.17 (0.48-2.84)
Interrupted sleeping												
No	146	18	1.00	154	10	1.00	151	13	1.00	157	7	1.00
Yes	518	85	1.49 (0.80-2.80)	575	28	0.67 (0.26-1.77)	585	18	0.28 (0.12-0.68)	586	17	0.54 (0.21-1.39)
Irritability or mood swings^b^												
No	270	24	1.00	283	11	1.00	281	13	1.00	282	12	1.00
Yes	393	79	2.19 (1.26-3.82)	445	27	1.20 (0.51-2.87)	454	18	0.79 (0.34-1.86)	460	12	0.59 (0.24-1.45)

**^a^**Adjusted for education, age at diagnosis, medical comorbidity, activity level within the past year of 30-month interview, a combined variable for menopause and hormone therapy, isoflavones from soy-containing foods. In addition, soy supplement, ginseng, flaxseed oil, black cohosh, yam, dong quai, red clover, licorice, alfalfa, DHEA, cat's claw, and other EBS use mutually adjusted. ^b^Excluded one woman with missing information of irritability or mood swings.

## Discussion

In the HEAL Study, neither overall EBS use nor the number of EBS types used was associated with HRQOL, fatigue, or hormone-related symptoms. Use of specific EBS types (soy supplements, ginseng, flaxseed oil, red clover, alfalfa, and DHEA) was associated with several outcomes of interest and we focus the discussion on these forms of EBS.

It is postulated that associations between soy supplements and breast cancer risk or progression may be in part related to the presence of isoflavones, which bind to estrogen receptors and activate estrogen response genes, although the hormone-like effect is much weaker than that of endogenous estradiol or estrone [43]. The estrogen-antagonist/-agonist effects of isoflavones may depend on a woman's endogenous estrogen levels or on the isoflavones concentration in the EBS compound. These compounds may function as estrogen agonists in women with low estrogen levels [44]. Results from clinical trials which have evaluated the impact of soy products on hot flashes are mixed; three showed no effect in breast cancer survivors [45-47] and one showed protective effects in postmenopausal women experiencing ≥ 5 hot flushes per day [48]. One randomized controlled trial observed that the incidence and severity of hot flashes were reduced two weeks after treatment with oral soy isoflavone extract, with no immediate reductions observed in the placebo group; the group differences achieved statistical significance at 6 weeks (*P *= 0.03), but decreased by 12 weeks (*P *= 0.08) [48]. Soy supplement use in the HEAL Study participants was associated with a better PCS score, but not with other outcomes examined. Our results suggest, on the whole, that soy supplements are unlikely to be detrimental to breast cancer survivors' HRQOL, fatigue, or hormone-related symptoms.

Ginsenosides (Rh1, Rb1, and Rg1) from ginseng have estrogen-like characteristics [49,50] and hence ginseng might ease menopausal symptoms. Ginseng did not influence hot flashes in a randomized, double blind, placebo-controlled study of ginseng in women reporting high frequency of hot flashes [51]. An observational study conducted in 2-5 years Chinese breast cancer survivors reported that ginseng use after cancer diagnosis, particularly current use, was positively associated with higher HRQOL scores in the psychological and social well-being domains, but was not associated with scores in the physical domain [52]. The findings from China may not translate to US populations because the major type of ginseng used could be different. An epidemiologic study conducted in US breast cancer survivors who were, on average, 6.5 years post diagnosis reported that ginseng users had lower SF-36 MCS scores [16]. We observed that ginseng use was associated with a lower PCS score, a higher fatigue score, and several hormone-related symptoms. Although these associations with different symptoms are consistent with previous studies outlining the adverse effects of ginseng [53-55], we cannot exclude the possibility that the symptoms experienced by women who took ginseng motivated their ginseng use.

Flaxseed oil is derived from the seeds of the flax plant that contain phytoestrogens and alpha-linolenic acid [56,57]. Colonic microflora convert phytoestrogens to enterolactone and enterodiol, both of which have estrogenic and antiestrogenic properties [58]. Alpha-linolenic acid had growth-inhibitory and proapoptotic effects on estrogen-positive breast cancer cells [59] and decreased the incidence, number, and growth of tumors in rats [60,61]. In human studies, flaxseed stabilized mood, improved depression symptoms [62], and reduced blood pressure during mental stress induced by frustrating cognitive tasks [63]. Our results are consistent with these findings as flaxseed oil was associated with higher MCS scores.

Red clover is another source of isoflavones, and had some efficacy in reducing hot flashes, but did not influence quality of life, in a 12-week randomized clinical trial [64]. In the HEAL Study, red clover use was associated with neither HRQOL nor hot flashes. However, red clover users were less likely to report three symptoms (weight gain, night sweats, and difficulty concentrating) than non-EBS users. Although these results support an association of red clover with fewer menopausal symptoms, it is important to note that we had only 38 users.

Alfalfa also contains phytoestrogens and has weak estrogenic effects [65,66]. In animal studies, alfalfa was associated with antioxidant activity [67] and protected against atherosclerotic lesions [68]. A small Italian study of women experiencing hot flashes and night sweats found that use of alfalfa and sage extracts for three months completely alleviated symptoms in 20 of 30 women studied [69]. In the HEAL Study, alfalfa users had a substantial but non-statistically-significant lower risk for hot flashes and were less likely to report interrupted sleep than non-EBS users. These results based on 31 alfalfa users provide some evidence that alfalfa may reduce menopausal symptoms.

DHEA is an endogenous steroid produced and secreted by the adrenal gland. Its sulfated form is converted into androgens and estrogens by specific steroidogenic enzymes. Blood DHEA levels begin to decrease around age 30, and by menopause are decreased 60%, on average [70]. It is reasonable to speculate that DHEA supplements may alleviate the symptoms caused by estrogen deficiency. A study that administered 50 mg of DHEA to 22 women found hot flash scores decreased 50% from baseline to week 5 of treatment [71]. In the HEAL Study, DHEA users had lower odds of hot flashes than non-EBS users. Our findings, based on 24 users, support that DHEA use may reduce hot flashes.

This study has several important limitations. First, the analysis relied on self-reported EBS use. Although our HRQOL and symptom data were collected, on average, 10 months after the information on EBS use that was collected, it is possible that the lower HRQOL or the symptoms experienced by women who took EBS were what motivated EBS use. This might bias our results towards the null value, underestimate the associations of EBS use with better health-related outcomes, or yield a false association of EBS use with poorer HRQOL or severe fatigue or other symptoms. Second, we were unable to rule out the possibility that some women might have changed their number or type of EBS they used, or non-EBS-users may have become users during an average of 10 month interval between our two surveys. If these events occurred, they would have biased our results toward the null, limiting our ability to detect associations with EBS use. Third, we did not collect some important information regarding EBS use such as when EBS use was initiated, duration or frequency of use, dosage level of supplements taken, or reasons for use. Furthermore, as this study is exploratory, we did not adjust for multiple comparisons. Clearly limitations restrict interpretation of observed associations. The results do provide preliminary information for future epidemiologic studies or clinical trials and add to the sparse literature on the association of EBS use with health-related outcomes.

We believe that this is the first epidemiologic analysis examining the potential association of overall EBS use, number of EBS types, and eleven commonly used types of EBS with multiple health-related outcomes. EBS use among breast cancer survivors is common, and data showing the efficacy of these agents (or lack thereof) on symptoms and HRQOL would be useful to survivors and their healthcare providers.

## Conclusions

Our results indicate the importance of assessing specific types of EBS separately in future efficacy studies since they may have distinct associations with health-related outcomes. The roles of soy supplements, flaxseed oil, red clover, alfalfa, and DHEA, in the improvement of HRQOL or alleviation of fatigue or hormone-related symptoms among breast cancer survivors merit further exploration.

## List of abbreviations

CAM: complementary/alternative medicines; CI: confidence interval; DHEA: dehydroepiandrosterone; EBS: estrogenic botanical supplement; FFQ: food-frequency questionnaire; HDIO: Herb-Drug Interactions in Oncology; HEAL: Health, Eating; Activity, and Lifestyle; HRQOL: health-related quality of life; HT: hormone therapy; MCS: mental component summary; NMCD: Natural Medicines Comprehensive Database; OR: odds ratio; SEER: Surveillance Epidemiology and End Results; PCS: physical component summary; PDR-H: Physician's Desk Reference for Herbal Medicines.

## Conflicts of interest statement

The authors declare that they have no competing interests.

## Authors' contributions

AM, KBB, RBB, and LB obtained funding for the HEAL Study, designed and implemented the HEAL surveys, and supervised data collection, data management, and data cleaning. HM conducted data analyses and drafted the manuscript. All authors participated in the revision of the manuscript and have read and approved the final version.

## Pre-publication history

The pre-publication history for this paper can be accessed here:

http://www.biomedcentral.com/1472-6882/11/109/prepub
